# Racial disparities in superficial venous disease management: A comparative study of interventions and patient-related outcomes

**DOI:** 10.1016/j.jvsv.2025.102363

**Published:** 2025-12-08

**Authors:** Garyn Metoyer, Ethan Chervonski, Giancarlo Speranza, Caron B. Rockman, Glenn R. Jacobowitz, Thomas S. Maldonado, Mikel Sadek

**Affiliations:** a Division of Vascular Surgery, New York University Langone Health, New York, NY; b Division of Vascular and Endovascular Surgery, University of Massachusetts Memorial Medical Center, Worcester, MA; c Division of Vascular Surgery, Hackensack University Medical Center, Hackensack, MA; d Division of Vascular Surgery, Northwell Health, New York, NY

**Keywords:** Procedural outcomes, Racial disparities, Venous insufficiency

## Abstract

**Objective:**

Chronic venous insufficiency (CVI) resulting in venous hypertension can cause lifestyle-limiting debilitation. Studies have identified racial and ethnic disparities in CVI presentation and clinical severity; however, there is limited literature examining disparities in CVI management and procedural outcomes among different racial and ethnic groups. The aim of this study was to characterize differences in endovenous treatment paradigms between racial and ethnic groups and to assess how this affected patient outcomes.

**Methods:**

The national Vascular Quality Initiative Varicose Vein Registry database was queried for superficial venous interventions, including endovenous radiofrequency ablation, endovenous laser ablation, high ligation, stripping, and microphlebectomy, performed from April 2014 to March 2024. We categorized patients as non-Hispanic White (NHW), non-Hispanic Black (NHB), Hispanic/Latino, Asian, and Other (including American Indian, Alaskan Native, Native Hawaiian, other Pacific Islander, more than one race, and unknown/other). Baseline demographics, clinical and treatment characteristics, complication rates, and changes in quality-of-life endpoints (ie, revised Venous Clinical Severity Score [rVCSS] and Heaviness, Achiness, Swelling, Throbbing, Itching [HASTI] score) were compared between racial/ethnic groups with NHW as the reference category. Linear regression and logistic regression/χ^2^ tests were used to compare continuous/ordinal and categorical variables, respectively.

**Results:**

A total of 65,090 superficial venous procedures encompassing endovenous thermal ablations, stripping/high ligation, and microphlebectomy were included. NHW patients underwent interventions for less severe baseline CVI based on CEAP class and had more superficial venous interventions (2.45 ± 1.95; *P* < .001) and repeat thermal ablations (1.66 ± 1.14; *P* < .001) than other groups. NHB had more severe baseline CVI based on higher prevalence of severe CEAP (ie, C5, C6, and C6r disease: 5.8%, 11.8%, and 0.9%, respectively; *P* < .05). NHB patients were less likely to have concomitant microphlebectomy than NHW (odds ratio, 0.79; 95% confidence interval, 0.73-0.87; *P* < .001). NHB had the highest rVCSS score preoperatively (8.17 ± 4.02; *P* < .001) with the largest improvement at ≤3 (−4.40 ± 5.23; *P* < .001) and >3 months (−7.00 ± 5.00; *P* < .001) following intervention. Hispanic/Latinos had the highest preoperative HASTI score (10.34 ± 5.40; *P* < .001) and the largest score reduction at ≤3 months (−6.62 ± 6.51; *P* < .001). Post procedure, Hispanics and other study groups were more likely to experience blistering and medication induced ulcer (*P* < .05). The other group was less likely to experience hematoma postoperatively (*P* < .05).

**Conclusions:**

This study highlights significant differences across racial/ethnic groups in the presentation, treatment and outcomes of patients with treated for CVI. Black patients undergo fewer ablations and superficial venous procedures overall; however, once treated, they exhibit significant symptomatic improvement.

**Article Highlights**

• **Type of Research:** Retrospective analysis of prospectively collected Vascular Quality Initiative data
• **Key Findings:** Among racial groups undergoing superficial venous interventions, Non-Hispanic White patients had significantly less severe chronic venous insufficiency and underwent more superficial treatments (2.45 ± 1.95) and repeat venous ablations (1.66 ± 1.14), whereas Non-Hispanic Black patients had more severe chronic venous insufficiency, fewer microphlebectomies, and significant improvement in quality of life assessments after treatment.
• **Take Home Message:** Potential disparities exist in venous insufficiency severity and treatments among patients in the Vascular Quality Initiative; racial and ethnic groups previously considered to face challenges with outcomes after venous ablation may instead show significant clinical improvement with intervention.

Estimated to affect around 25% of the United States population, chronic venous insufficiency (CVI) of the lower extremities is a common vascular disorder, resulting in a range of aesthetic and clinical manifestations.^1^^,^^2^ The pathophysiology of CVI is multifactorial, involving valvular dysfunction, venous obstruction, and systemic factors like obesity and heart failure, which can lead to reflux and venous hypertension. CVI symptoms may progress to quality-of-life impairment causing pain, discomfort, ulceration, and functional limitations. Several clinical and patient reported assessment tools exist to categorize the severity of CVI, including the revised Venous Clinical Severity Score (rVCSS), Clinical, Etiology, Anatomy and Pathology (CEAP) classification, Heaviness, Achiness, Swelling, Throbbing, Itching (HASTI) score, Chronic Venous Insufficiency Quality of Life Questionnaire 20 (CIVIQ-20), VVSymQ, and so on.^3^, ^4^, ^5^ The literature has identified racial disparities in symptom presentation and clinical severity of CVI, noting that racial and ethnic minority groups often present with more severe forms of CVI at initial diagnosis and may experience delays in seeking and receiving care.^6^

The American Venous Forum and Society for Vascular Surgery clinical practice guidelines recommend against compression therapy as the primary treatment of symptomatic CVI for patients eligible for vein ablation.^7^ Thermal and nonthermal vein ablative therapies are widely used and highly effective treatments for CVI, with recent updates emphasizing endovenous ablation over open surgical techniques to reduce post-procedural pain and morbidity and allow for an earlier return to regular activity.^2^^,^^7^ Varicose tributaries and tortuous superficial truncal veins may be removed by adjunctive microphlebectomy in either staged or concomitant approaches.^7^ Despite the prevalence of CVI across diverse populations, there are limited data that quantify the severity of CVI among affected individuals and the results following procedural treatment.^8^

This lack of literature highlights a gap in understanding of disparities that may exist in the context of CVI diagnosis, treatment, and outcomes. By querying the national Vascular Quality Initiative (VQI) Varicose Vein Registry (VVR) database, this study aims to evaluate the use of endovenous ablation for symptomatic CVI across different racial and ethnic groups to identify severity at disease presentation, patterns in treatment, and patient outcomes.

## Methods

The national VQI VVR was queried for superficial venous procedures, including thermal endovenous ablations (ie, radiofrequency or laser ablation) and open surgical interventions (ie, stripping, high ligation, and microphlebectomy), performed from April 2014 to March 2024. Nonthermal endovenous ablations, those without a patient’s race/ethnicity recorded, and those under 18 years of age were excluded. The use of the VQI VVR was granted after submission and approval of the scientific protocol through the national VQI application process, and Institutional Review Board exemption was obtained for evaluation of the national VQI registry data. All patient information was deidentified and did not require informed consent. Once access was granted, the data set of all procedures in the VQI VVR for the listed dates was made available. Patients were categorized by racial and ethnic group into the following groups: non-Hispanic White (NHW), non-Hispanic Black (NHB), Hispanic/Latino, Asian, and Other (including American Indian, Alaskan Native, Native Hawaiian, other Pacific Islander, more than 1 race, and unknown/other). Baseline demographics, clinical and procedural/treatment characteristics, post-procedural complication rates, and changes in clinical and patient reported quality-of-life endpoints (rVCSS and HASTI scores) were compared between racial and ethnic groups with NHW serving as the reference category.

The primary outcome was the clinical severity of CVI among racial and ethnic groups according to standardized patient- and physician-reported scoring systems, including CEAP, HASTI, and rVCSS, at the time of intervention. Secondary outcomes included the change in HASTI and rVCSS following intervention, the average number of total superficial venous procedures performed in a given patient, the average number of repeat thermal ablations performed in a given patient (among those who underwent at least one thermal ablation), and the rate of concomitant microphlebectomy with a given axial vein procedure. The change in quality-of-life endpoints were measured at the latest available follow-up within 3 months and after 3 months of the procedure. Additionally, postoperative complications were recorded. These included perioperative systemic complications and postprocedural hematologic and dermatologic complications. For variables with missing data points, percentages were calculated, and statistical tests were performed among those patients with available data. All outcomes were measured on a per-procedure basis, rather than per-patient basis. Clarification of variable definitions and the patient-reported symptom scale are provided in the Supplementary Methods (online only).

### Statistical analysis

Continuous and ordinal variables were presented as means with standard deviations and compared between NHW (reference category) and other racial/ethnic groups using linear regression. Categorical variables were presented as numbers with percentages of the relevant populations and compared between NHW (reference category) and other racial/ethnic groups using logistic regression. Odds ratios (ORs) with 95% confidence intervals (CIs) were shown for logical regression. Additionally, pairwise χ^2^ tests were performed to compare the distribution of CEAP clinical classes and insurance status between NHW and each other racial/ethnic group. All statistical tests were two-sided, and a *P*-value < .05 was considered statistically significant. Analyses were performed using SPSS Statistics, version 30.0.0.0 (IBM Inc). Manuscript preparation was done in accordance with the REporting of studies Conducted using Observational Routinely collected health Data (RECORD) checklist.^9^

## Results

A total of 65,090 superficial venous procedures encompassing endovenous thermal ablations, stripping/high ligation, and microphlebectomy were included. Baseline demographics, clinical characteristics, and CEAP clinical classifications were collected (Table I). Our study results show that Black patients with CVI are more likely to be female; have higher body mass index; have Medicaid or Miliary and Veteran Affairs insurance (*P* < .05), or pay out of pocket; are more likely to be younger, and are more likely to have a history of deep vein thrombosis compared with NHW (*P* < .001). In comparison to other racial and ethnic groups studied, NHB had a disproportionately higher prevalence of severe CEAP clinical classifications based on the proportion of C5, C6, and C6r disease (5.8%, 11.8%, and 0.9%, respectively; *P* < .05).

**Table I:** Baseline demographics, clinical features, treatment characteristics, and Clinical signs, Etiology, Anatomy, and Pathophysiology (*CEAP*) clinical class by race/ethnicity

	NHW (n = 50,221)	NHB (n = 2362)	Hispanic/Latino (n = 6205)	Asian (n = 1040)	Other^a^ (n = 5262)
Demographics					
Age, years	56.3 ± 14.2	55.2 ± 12.9^b^	52.9 ± 13.5^b^	55.2 ± 14.3^c^	54.3 ± 14.0^b^
Female sex at birth	33,878 (67.5)	1542 (65.3)^c^ OR, 0.91; 95 % CI, 0.83-0.99	4587 (73.9)^b^ OR, 1.37; 95% CI, 1.29-1.45	674 (64.8) OR, 0.89; 95% CI, 0.78-1.01	3625 (68.9)^c^ OR, 1.07; 95% CI, 1.01-1.14
Insurance status					
Medicare	11,573 (23.1)	426 (18.0)^b^	704 (11.4)^b^	148 (14.3)^b^	963 (18.3)^b^
Medicaid	2542 (5.1)	291 (12.3)^b^	734 (11.9)^b^	64 (6.2)	330 (6.3)^b^
Commercial	33,872 (67.5)	1471 (62.3)^b^	4381 (70.7)^b^	745 (71.8)^d^	3804 (72.3)^b^
Military and Veteran Affairs	624 (1.2)	43 (1.8)^c^	52 (0.8)^d^	17 (1.6)	73 (1.4)
Foreign insurance	4 (0.008)	4 (0.2)^b^	3 (0.05)^d^	0 (0.0)	8 (0.2)^b^
Self-pay	374 (0.7)	81 (3.4)^b^	281 (4.5)^b^	16 (1.5)^d^	48 (0.9)
Medicare Advantage	1208 (2.4)	46 (1.9)	39 (0.6)^b^	48 (4.6)^b^	34 (0.6)^b^
Clinical characteristics					
Body mass index, kg/m^2^	29.9 ± 7.2	33.8 ± 8.0^b^	30.1 ± 6.5^c^	25.3 ± 4.7^b^	28.5 ± 6.7^b^
History of deep venous thrombosis	3798 (7.6)	259 (11.0)^b^ OR, 1.51; 95% CI, 1.32-1.72	366 (5.9)^b^ OR, 0.77; 95% CI, 0.69-0.86	27 (2.6)^b^ OR, 0.33; 95% CI, 0.22-0.48	298 (5.7)^b^ OR, 0.73; 95% CI, 0.65-0.83
History of pulmonary embolism	550 (1.1)	32 (1.4) OR, 1.24; 95% CI, 0.87-1.78	47 (0.8)^c^ OR, 0.69; 95% CI, 0.51-0.93	7 (0.7) OR, 0.61; 95% CI, 0.29-1.29	16 (0.3)^b^ OR, 0.28; 95% CI, 0.17-0.45
History of superficial phlebitis	5855 (11.7)	287 (12.2) OR, 1.05; 955 CI, 0.92-1.19	541 (8.7)^b^ OR, 0.72; 95% CI, 0.66-0.79	76 (7.3)^b^ OR, 0.60; 95% CI, 0.47-0.76	532 (10.1)^b^ OR, 0.85; 95% CI, 0.78-0.94
Treatment characteristics					
No. superficial venous interventions per patient	2.45 ± 1.95	1.79 ± 1.11^b^	2.03 ± 1.49^b^	1.57 ± 0.77^b^	2.26 ± 1.85^b^
No. thermal ablations per patient with at least one ablation	1.66 ± 1.14	1.39 ± 0.75^b^	1.52 ± 0.88^b^	1.31 ± 0.59^b^	1.52 ± 1.06^b^
Concomitant microphlebectomy (with thermal ablation)	20,374 (40.6)	830 (35.1)^b^ OR, 0.79; 95% CI, 0.73-0.87	3909 (63.0)^b^ OR, 2.49; 95% CI, 2.36-2.63	492 (47.3)^b^ OR, 1.32; 95% CI, 1.16-1.49	2125 (40.4) OR, 0.99; 95% CI, 0.94-1.05
CEAP classification					
C0	119 (0.2)	8 (0.4)	7 (0.1)	2 (0.2)	18 (0.3)
C1	286 (0.6)	9 (0.4)	61 (1.0)^b^	0 (0.0)^c^	15 (0.3)^d^
C2	16,785 (33.9)	505 (22.3)^b^	2406 (39.3)^b^	416 (43.2)^b^	2129 (41.1)^c^
C2r	326 (0.7)	11 (0.5)	30 (0.5)	6 (0.6)	25 (0.5)
C3	18,532 (37.4)	810 (35.7)	1486 (24.3)^b^	271 (28.2)^b^	1545 (29.8)^b^
C4a	7790 (15.7)	423 (18.7)^b^	1635 (26.7)^b^	175 (18.2)^c^	1057 (20.4)^b^
C4b	1521 (3.1)	79 (3.5)	116 (1.9)^b^	23 (2.4)	75 (1.4)^b^
C4c	58 (0.1)	1 (0.04)	6 (0.1)	12 (1.2)^b^	13 (0.3)^c^
C5	1381 (2.8)	132 (5.8)^b^	139 (2.3)^c^	21 (2.2)	113 (2.2)^c^
C6	2661 (5.4)	268 (11.8)^b^	223 (3.6)^b^	35 (3.6)^c^	184 (3.6)^b^
C6r	84 (0.2)	21 (0.9)^b^	7 (0.1)	1 (0.1)	11 (0.2)

*CI,* Confidence interval; *NHB,* non-Hispanic Black/African American; *NHW,* non-Hispanic White; *OR,* odds ratio.

Data are presented as number (%) or mean ± standard deviation.

Based on logistic regression, linear regression, or pairwise χ^2^ tests (only for CEAP) comparing NHW (reference category) with each other racial/ethnic group.

a Includes American Indian, Alaskan Native, Native Hawaiian, other Pacific Islander, more than one race, and unknown/other.

b *P* < .001.

c *P* < .05.

d *P* < .01.

Perioperative quality-of-life data are shown in Table II. Among different racial and ethnic groups, NHW patients had more superficial venous interventions (2.45 ± 1.95; *P* < .001) and repeat thermal ablations (1.66 ± 1.14; *P* < .001). NHB had higher preoperative rVCSS (8.17 ± 4.02; *P* < .001) and HASTI scores (9.70 ± 5.55; *P* < .001). NHB patients were less likely to have concomitant microphlebectomy than NHW (OR, 0.79; 95% CI, 0.73;0.87; *P* < .001). Black patients who underwent a superficial venous intervention had greater improvement in rVCSS and HASTI scores demonstrated at both short-term (rVCSS: −4.40 ± 5.23; *P* < .001; HASTI: −6.56 ± 7.13; *P* < .01) and long-term (rVCSS: −7.00 ± 5.00; *P* < .001; HASTI: −8.93 ± 6.02; *P* < .001) follow-up intervals. Hispanic/Latinos had the highest preoperative HASTI score (10.34 ± 5.40; *P* < .001) and the largest score improvement at ≤3 months (−6.62 ± 6.51; *P* < .001).

**Table II:** Quality of life after superficial venous intervention by race and ethnicity

	NHW (reference category)	NHB	Hispanic/Latino	Asian	Other^a^
Preoperative quality-of-life metrics					
VCSS	7.35 ± 3.24	8.17 ± 4.02^b^	7.44 ± 3.09^c^	6.84 ± 3.21^b^	7.62 ± 3.68^b^
HASTI score	8.60 ± 5.19	9.70 ± 5.55^b^	10.34 ± 5.40^b^	8.92 ± 5.58	9.89 ± 5.28^b^
Postoperative quality-of-life metrics at early follow-up (≤3 months)					
VCSS	3.91 ± 3.50	3.73 ± 3.34	3.79 ± 3.38	3.89 ± 3.26	4.01 ± 3.61
VCSS change from baseline	−3.36 ± 4.86	−4.40 ± 5.23^b^	−3.38 ± 4.53	−2.63 ± 3.93^c^	−3.55 ± 5.20
HASTI score	3.13 ± 4.05	3.08 ± 4.21	2.83 ± 3.71^c^	3.29 ± 4.05	3.28 ± 4.24
HASTI change from baseline	−5.51 ± 6.64	−6.56 ± 7.13^d^	−6.62 ± 6.51^b^	−5.38 ± 6.60	−6.51 ± 6.85^b^
Postoperative quality-of-life metrics at late follow-up (>3 months)					
VCSS	1.30 ± 2.69	1.18 ± 2.57	1.32 ± 2.57	1.10 ± 2.45	1.20 ± 2.59
VCSS change from baseline	−5.88 ± 4.34	−7.00 ± 5.00^b^	−5.84 ± 3.98	−5.33 ± 3.82	−6.21 ± 4.60^d^
HASTI score	0.89 ± 2.57	0.82 ± 2.53	0.91 ± 2.53	0.74 ± 2.51	0.82 ± 2.51
HASTI change from baseline	−7.82 ± 5.88	−8.93 ± 6.02^b^	−8.71 ± 5.77^b^	−7.59 ± 6.69	−8.73 ± 5.78^b^

*HASTI,* Heaviness, Achiness, Swelling, Throbbing, Itching; *NHB,* non-Hispanic Black/African American; *NHW,* non-Hispanic White; *VCSS,* Venous Clinical Severity Score.

Data are presented as mean ± standard deviation.

Based on linear regression analysis comparing NHW (reference category) with each other racial/ethnic group.

a Includes American Indian, Alaskan Native, Native Hawaiian, other Pacific Islander, more than one race, and unknown/other.

b *P* < .001.

c *P* < .05.

d *P* < .01.

Among complications, Hispanics and Other group were more likely to experience blistering and medication-induced ulcers postoperatively (*P* < .05). The Other group was less likely to experience hematoma postoperatively (*P* < .05) (Supplementary Tables I-IV, online only).

## Discussion

Our study results suggest that, although Black patients may have more advanced disease at the time of treatment, they also derive significant clinical and quality-of-life benefits from the procedure, with fewer procedures being performed, which may be impacted by patient selection, location of intervention, and postoperative care.^10^ We also found that Asian patients undergoing thermal ablations for CVI also had fewer procedures, but they had less pronounced improvements in post-procedural outcomes. As similar results have been published, potential contributing factors to our findings may include lack of preventative care among Asian patients.^11^^,^^12^ Differences in vein anatomy, reflux patterns, and age-independent factors among Asian patients may also contribute to our study results.^13^^,^^14^ Further studies are needed to evaluate these differences.

Similar large-scale studies performed by Pappas et al and Chan et al analyzed racial disparities in endovenous procedures including thermal ablation and vein stripping among patients with CVI, using private institutional data and the National Surgical Quality Improvement Program (NSQIP).^8^^,^^11^ Our study results differ from these studies in several ways. Pappas et al analyzed 20,644 patients who underwent endovenous procedures, and they found that the Hispanic study population (1.98 ± 1.24) and Others (2.07 ± 1.25) required fewer stand-alone ablations compared with Whites (2.31 ± 1.56), Asians (2.36 ± 1.58), and African Americans (2.27 ± 1.56; *P* < .0001), which differs from our study results. Pappas et al also found that, for patients receiving standalone ablation, the White study population had higher preintervention rVCSS values compared with other races, and among patients that underwent ablation and phlebectomy, the Hispanic study population demonstrated the greatest degree of improvement among racial and ethnic groups. This differs from our results that showed the Black study population had a higher rVCSS and a greater degree of improvement at short-term (<3 months) and long-term (>3 months) follow-up compared with the White study population. Pappas et al used data derived from a specialized outpatient clinical setting, which may not represent the broader population of patients with CVI as captured by the VQI VVR and may influence treatment outcomes and disease severity assessments. Comparatively, their study population had a much larger proportion of patients undergoing intervention with CVI classified as CEAP C0, C1, C2, C3 and lower preoperative rVCSS scores across all racial demographics. Their length of follow-up to assess postprocedural rVCSS was 1 month, which may limit time for observation of improvement following intervention, as compared with the longer follow-up data provided by this study.^15^

Chan et al performed an analysis of 21,025 patients that underwent vein stripping and radiofrequency and laser ablation to analyze temporal trends of these venous treatments and identify disparities among racial groups for undergoing ablation compared with vein stripping from 2011 to 2018 in the NSQIP.^11^ Based on inherent differences between databases, VQI VVR offers advantages in incorporating venous outcome-specific data pertinent to our study over NSQIP, which lacks information regarding preoperative venous disease severity and improvement following intervention as literature has demonstrated poor concordance for most postoperative variables when comparing the NSQIP with the VQI VVR.^16^^,^^17^ The comprehensiveness of the VQI database helps strengthen the generalizability of this study based on the extensive patient and procedural data provided and the diverse collective of health care facilities included, which are critical to the analysis of this study. These elements enhance the relevance and applicability of this study’s results, making them valuable for informing providers and further research study.

Zil-E-Ali et al similarly analyzed the VQI database for patients that underwent ablation of the great saphenous vein only and excluded patients with other vein segments or multiple segments.^18^ They analyzed 9009 patients, comparing CEAP, rVCSS, and patient-reported outcomes (PROs) (ie, HASTI scores) among racial and ethnic groups before and after intervention. Contrary to our study, their Black/African American study group had the least improvement in pain and varicose veins compared with the Hispanic and White study groups (Black/African American, 3.52 ± 4.4; Hispanic, 4.42 ± 3.78; White, 4.25 ± 4.13; *P* < .001). Comparing PROs, Black/African American patients had the least improvement (6.64 ± 8.3) relative to the White (8.82 ± 8.0) and Hispanic patients (8.95 ± 9.1; *P* < .001). Their Black/African American patient study group disproportionately had the largest proportion of CEAP class C5 and C6 disease (+4.7% and +12.7%, respectively; *P* < .001 and the smallest increase in proportion within the C1 category post procedure (+33.9%). Meanwhile, their Hispanic patients had the largest improvement to C1 category (+57.1%) compared with the other races. These findings are different from the rVCSS and HASTI scores seen in our study. To compare the differences, their study limited inclusion to patients who had great saphenous vein ablation only, which may not capture the full complexity of this patient population. Also, the anatomical component of CEAP classification was updated in 2020, adding anatomical terms for the classification of vein segments, and this was added to our study.^19^ Our study also provides a more updated query of the VQI registry. According to these study results, despite more severe CVI, Black patients undergo significantly fewer thermal ablations and superficial venous procedures overall including microphlebectomy than the White patient population. These study results emphasize a potential disparity that may be associated with the increased rates of infections, need for advanced wound care, and increased hospital admissions among the Black patients with CVI.^20^^,^^21^ Potential factors contributing to the observed study results include differences in insurance coverage for venous procedures, referral patterns to specialists for evaluation, and potential mistrust in the medical system.^11^^,^^13^^,^^22^, ^23^, ^24^ Our study results highlight disparities in the periprocedural, procedural, and outcomes related to the delivery of care for patients with CVI. One possible intervention includes improving education and outreach for primary-care providers on the diagnosis and management of CVI, as these clinicians are often on the front lines.^23^ This should be coupled when appropriate with prompt referral to a venous specialist, improving the insurance milieu, and more intensive management for patients with venous ulcers.^25^

Regarding insurance coverage, NHB and Hispanics rely on Medicaid significantly more than NHW, and they also have higher rates of being uninsured, reflecting possible socioeconomic differences and disparities in health care access. Pappas et al, while assessing comparative outcomes for the treatment of CVI in Medicare- and non Medicare-eligible patients, similarly demonstrated disparities in insurance status, with a higher proportion of Hispanic and African-American patients relying on Medicaid for access to venous ablation, and Hispanic patients more likely paying out-of-pocket, although statistical analysis was not included to assess statistical significance.^13^ Our study adds an important perspective, providing data from the VQI registry to contribute to these notable trends in insurance status across a broad spectrum of health care delivery for patients undergoing endovenous ablation and demonstrate potential disparities that may contribute to the high burden of disease among underserved communities and the subsequently large health care expenditure needed to treat severe manifestations of CVI for these patients.^6^

Our study has several limitations. VQI is a database of patients who undergo treatment, and this study cannot extrapolate the burden of superficial venous insufficiency among the larger United States population who do not receive treatment or receive treatment outside of the VQI registry. As such, racial and ethnic groups may be overrepresented or underrepresented in our study results, considering baseline population size among different groups. Considering potential discordance between external physical exam and symptoms, skin tone could play a role in treatment of patients with C1 and C2 disease, leading to detection bias.^26^ The data in the VQI database spans multiple years, during which updates to clinical practice guidelines have been published by the American Venous Forum.^7^^,^^15^ These temporal changes can influence treatment patterns and subsequent outcomes and are confounding variables in the analysis of our study results. Lastly, our study excluded the nonthermal ablation techniques, potentially underestimating the clinical impact of nonthermal ablations on patient outcomes. We chose to exclude nonthermal ablations as reported studies have conflicting results regarding superiority of ablation modalities.^27^, ^28^, ^29^ Also considering the novelty of cyanoacrylate glue and other nonthermal ablation techniques, potential variability in use and access to treatment and inconsistent practice protocols acting as confounders, we excluded nonthermal techniques.^30^, ^31^, ^32^ However, as adoption of these techniques continues to increase over time and randomized control trials describe comparable efficaciousness, future studies will be needed to analyze if potential disparities exist among patient populations.^33^^,^^34^ Despite these limitations, our study leverages the comprehensiveness and detail of the VQI registry to demonstrate potential disparities in severity of venous insufficiency and endovenous treatments among the VQI registry patient population and demonstrate clinical improvement among racial and ethnic groups previously thought to have challenges with quality-of-life and clinical outcomes following ablation.^8^ Provider bias and population and cultural differences are, in ways, inherent to disparities in research study and difficult to exclude, as data at the individual level is not collected through the VQI registry^35^; however, this study fosters future research to investigate barriers in diagnosis, treatment access, and provider practices while promoting awareness of disparities, encouraging development of population-based approaches that assess gaps in care delivery, and advocating for standardized, bias-minimizing methods of disease severity assessment.

## Conclusions

In conclusion, this study highlights significant differences across racial and ethnic groups in the presentation, treatment and outcomes of CVI, underscoring the need for targeted education for patients, providers, and proceduralists and advocacy to insurance companies to improve care of patients burdened with CVI.

## Author Contributions

Conception and design: GM, MS, TM, CR, GJ

Analysis and interpretation: GM, EC, SG

Data collection: Not applicable

Writing the article: GM, EC, SG

Critical revision of the article: GM, MS, TM, EC, SG, CR, GJ

Final approval of the article: GM, MS, TM, EC, SG, CR, GJ

Statistical analysis: EC

Obtained funding: Not applicable

Overall responsibility: GM

## Funding

None.

## Disclosure

M.S. is a consultant for Boston Scientific and BD. The remaining authors report no conflicts.
