# Supplementary material for: Racial disparities in superficial venous disease management: A comparative study of interventions and patient-related outcomes
Source: J Vasc Surg Venous Lymphat Disord. 2025 Dec 8;14(2):102363. doi: 10.1016/j.jvsv.2025.102363 (PMC12870761; doi:10.1016/j.jvsv.2025.102363)
Supplement: Supplementary Tables [file mmc1.docx]

**Supplement Table I: Periprocedural Systemic Complications by Race/Ethnicity**

|  | Non-Hispanic White (N=50221) | Non-Hispanic Black (N=2362) | Hispanic/Latino (N=6205) | | | Asian (N=1040) | Other (N=5262) | | |
| --- | --- | --- | --- | --- | --- | --- | --- | --- | --- |
| Composite | 136 (0.27%) | 1 (0.04%)  OR 0.16 [0.022, 1.12] | | 10 (0.16%)  OR 0.59 [0.31, 1.13] | | 4 (0.38%)  OR 1.42 [0.52, 3.85] | 9 (0.17%)  OR 0.63 [0.32, 1.24] | | |
| Mild allergic reaction | 22 (0.044%) | 0 (0.0%)  OR 1.4e-6 [0.0,-] | 1 (0.016%)  OR 0.37 [0.050, 2.73] | | | 0 (0.0%)  OR 1.4e-6 [0.0,-] | 0 (0.0%)  OR 1.4e-6 [0.0,-] | | |
| Severe allergic reaction | 6 (0.012%) | 0 (0.0%)  OR 5.2e-6 [0.0,-] | | | 0 (0.0%)  OR 5.2e-6 [0.0,-] | 0 (0.0%)  OR 5.2e-6 [0.0,-] | | 0 (0.0%)  OR 5.2e-6 [0.0,-] |  |
| Migraine | 9 (0.018%) | 0 (0.0%)  OR 3.5e-6 [0.0,-] | | | 2 (0.032%)  OR 1.80 [0.39, 8.33] | 0 (0.0%)  OR 3.5e-6 [0.0,-] | | 0 (0.0%)  OR 3.5e-6 [0.0,-] |  |
| Visual disturbance | 11 (0.022%) | 0 (0.0%)  OR 2.8e-6 [0.0,-] | | | 1 (0.016%)  OR 0.74 [0.095, 5.70] | 0 (0.0%)  OR 2.8e-6 [0.0,-] | | 0 (0.0%)  OR 2.8e-6 [0.0,-] |  |
| Cough/chest tightness | 4 (0.0080%) | 0 (0.0%)  OR 7.8e-6 [0.0,-] | | | 1 (0.016%)  OR 2.02 [0.23, 18.11] | 0 (0.0%)  OR 7.8e-6 [0.0,-] | | 0 (0.0%)  OR 7.8e-6 [0.0,-] |  |
| Systemic infection | 4 (0.0080%) | 0 (0.0%)  OR 7.8e-6 [0.0,-] | | | 0 (0.0%)  OR 7.8e-6 [0.0,-] | 0 (0.0%)  OR 7.8e-6 [0.0,-] | | 0 (0.0%)  OR 7.8e-6 [0.0,-] |  |
| Transient ischemic attack | 1 (0.0020%) | 0/2362 (0.0%)  OR 3.1e-5 [0.0,-] | | | 0 (0.0%)  OR 3.1e-5 [0.0,-] | 0 (0.0%)  OR 3.1e-5 [0.0,-] | | 0 (0.0%)  OR 3.1e-5 [0.0,-] |  |
| Stroke | 1 (0.0020%) | 0 (0.0%)  OR 3.1e-5 [0.0,-] | | | 0 (0.0%)  OR 3.1e-5 [0.0,-] | 0 (0.0%)  OR 3.1e-5 [0.0,-] | | 0 (0.0%)  OR 3.1e-5 [0.0,-] |  |
| Pulmonary embolism | 7 (0.014%) | 0 (0.0%)  OR 4.4e-6 [0.0,-] | | | 0 (0.0%)  OR 4.4e-6 [0.0,-] | 0 (0.0%)  OR 4.4e-6 [0.0,-] | | 0 (0.0%)  OR 4.4e-6 [0.0,-] |  |
| Death | 0 | 0 (0.0%) | | | 0 (0.0%) | 0 (0.0%) | | 0 (0.0%) |  |
| Other Complication | 77 (0.15%) | 1 (0.042%)  OR 0.29 [0.040, 2.07] | | | 7 (0.11%)  OR 0.77 [0.35, 1.66] | 4 (0.38%)  OR 2.62 [0.96, 7.17] | | 9 (0.17%)  OR 1.16 [0.58, 2.32] |  |
| Values represent No. (%) or Mean±SD  Based on logistic regression, linear regression, or pairwise chi-squared tests (only for CEAP) comparing NHW (reference category) with each other racial/ethnic group:  *p<0.05  † p<0.01  ‡ p<0.001  ** Includes American Indian, Alaskan Native, Native Hawaiian, other Pacific Islander, more than one race, and unknown/other | | | | | | | | |  |

**Supplement Table II: Post-procedural Limb-specific Hematological Complications by Race/Ethnicity**

|  | Non-Hispanic White (N=25852) | Non-Hispanic Black (N=1338) | Hispanic/Latino (N=3004) | Asian (N=449) | Other (N=3188) |
| --- | --- | --- | --- | --- | --- |
| Composite | 450 (1.7%) | 26 (1.9%)  OR 1.12 [0.75, 1.67] | 44 (1.5%)  OR 0.84 [0.61, 1.15] | 9 (2.0%)  OR 1.16 [0.59, 2.25] | 46 (1.4%)  OR 0.83 [0.61, 1.12] |
| Deep vein thrombosis | 125 (0.48%) | 7 (0.52%)  OR 1.08 [0.51, 2.32] | 10 (0.33%)  OR 0.68 [0.36, 1.31] | 1 (0.22%)  OR 0.46 [0.064, 3.29] | 11 (0.35%)  OR 0.71 [0.38, 1.32] |
| Superficial phlebitis | 219 (0.85%) | 16 (1.20%)  OR 1.42 [0.85, 2.36] | 22 (0.73%)  OR 0.86 [0.56, 1.34] | 5 (1.11%)  OR 1.32 [0.54, 3.21] | 28 (0.88%)  OR 1.04 [0.70, 1.54] |
| Bleeding requiring intervention | 6 (0.023%) | 1 (0.075%)  OR 3.22 [0.39 26.78] | 0 (0.0%)  OR 2.7e-6 [0.0, -] | 0 (0.0%)  OR 2.7e-6 [0.0, -] | 1 (0.031%)  OR 1.35 [0.16, 11.23] |
| Hematoma | 111 (0.43%) | 6 (0.45%)  OR 1.05 [0.46, 2.38] | 14 (0.47%)  OR 1.09 [0.62, 1.90] | 3 (0.67%)  OR 1.56 [0.49, 4.93] | **6 (0.19%)***  **OR 0.44 [0.19, 0.995]** |
| Values represent No. (%) or Mean±SD  Based on logistic regression, linear regression, or pairwise chi-squared tests (only for CEAP) comparing NHW (reference category) with each other racial/ethnic group:  *p<0.05  † p<0.01  ‡ p<0.001  ** Includes American Indian, Alaskan Native, Native Hawaiian, other Pacific Islander, more than one race, and unknown/other | | | | | |

**Supplement Table III: Proximal thrombus extension among endovenous thermal ablations by Race/Ethnicity**

|  | Non-Hispanic White (N=25852) | Non-Hispanic Black (N=1338) | Hispanic/Latino (N=3004) | Asian (N=449) | Other (3188) |
| --- | --- | --- | --- | --- | --- |
| Proximal thrombus extension | 181 (0.70%) | 9 (0.67%)  OR 0.96 [0.49,1.88] | 26 (0.87%)  OR 1.24 [0.82, 1.87] | 3 (0.67%)  OR 0.95 [0.30, 3.00] | 31 (0.97%)  OR 1.39 [0.95, 2.04] |
| Endothermal heat-induced thrombosis (EHIT) II-IV | 70 (0.27%) | 1 (0.07%)  OR 0.28 [0.038, 1.99] | 9 (0.30%)  OR 1.11 [0.55, 2.22] | 1 (0.22%)  OR 0.82 [0.11, 5.93] | 9 (0.28%)  OR 1.04 [0.52, 2.09] |
| Values represent No. (%) or Mean±SD  Based on logistic regression, linear regression, or pairwise chi-squared tests (only for CEAP) comparing NHW (reference category) with each other racial/ethnic group:  *p<0.05  † p<0.01  ‡ p<0.001  ** Includes American Indian, Alaskan Native, Native Hawaiian, other Pacific Islander, more than one race, and unknown/other | | | | | |

**Supplement Table IV: Post-procedural Limb-specific Dermatologic Complications by Race/Ethnicity**

|  | Non-Hispanic White (N=25852) | Non-Hispanic Black (N=1338) | Hispanic/Latino (N=3004) | Asian (N=449) | Other (N=3188) |
| --- | --- | --- | --- | --- | --- |
| Composite | 452 (1.7%) | 26 (1.9%)  OR 1.11 [0.75, 1.66] | 66 (2.2%)  OR 1.26 [0.97, 1.64] | 9 (2.0%)  OR 1.15 [0.59, 2.24] | **60 (1.9%)**  OR 1.08 [0.82, 1.42] |
| Blister skin | 51 (0.20%) | 6 (0.45%)  OR 2.28 [0.98, 5.32] | **13 (0.43%)***  **OR 2.20 [1.20, 4.05]** | 1 (0.22%)  OR 1.13 [0.16, 8.19] | **15 (0.47%)^†^**  **OR 2.39 [1.34, 4.26]** |
| Paresthesia | 220 (0.85%) | 7 (0.52%)  OR 0.61 [0.29, 1.30] | 26 (0.87%)  OR 1.02 [0.68, 1.53] | 3 (0.67%)  OR 0.78 [0.25, 2.46] | 26 (0.82%)  OR 0.96 [0.64, 1.44] |
| Pigmentation | 139 (0.54%) | 11 (0.82%)  OR 1.53 [0.83, 2.84] | 20 (0.67%)  OR 1.24 [0.78, 1.98] | 4 (0.89%)  OR 1.66 [0.61, 4.51] | 12 (0.38%)  OR 0.70 [0.39, 1.26] |
| Medication-induced ulcer | 5 (0.019%) | 1 (0.075%)  OR 3.87 [0.45, 33.12] | 1 (0.033%)  OR 1.72 [0.20, 14.74] | 0 (0.0%)  OR 3.2e-6 [0.0, -] | **3 (0.094%)***  **OR 4.87 [1.16, 20.38]** |
| Wound infection | 43 (0.17%) | 1 (0.07%)  OR 0.45 [0.062, 3.26] | 6 (0.20%)  OR 1.20 [0.51, 2.82] | 1 (0.22%)  OR 1.34 [0.18, 9.75] | 8 (0.25%)  OR 1.51 [0.71, 3.21] |
| Values represent No. (%) or Mean±SD  Based on logistic regression, linear regression, or pairwise chi-squared tests (only for CEAP) comparing NHW (reference category) with each other racial/ethnic group:  *p<0.05  † p<0.01  ‡ p<0.001  ** Includes American Indian, Alaskan Native, Native Hawaiian, other Pacific Islander, more than one race, and unknown/other | | | | | |
