# Supplementary material for: Racial disparities in superficial venous disease management: A comparative study of interventions and patient-related outcomes
Source: J Vasc Surg Venous Lymphat Disord. 2025 Dec 8;14(2):102363. doi: 10.1016/j.jvsv.2025.102363 (PMC12870761; doi:10.1016/j.jvsv.2025.102363)
Supplement: Supplementary Material [file mmc2.docx]

**SUPPLEMENTARY METHODS (E-Appendix):**

*Abbreviated Heaviness, Achiness, Swelling, Throbbing, Itching (HASTI) Symptom Score*

The abbreviated HASTI symptom score was derived from five symptom domains—heaviness, achiness, swelling, throbbing, and itching—each rated on a 6-point scale: 0 = none of the time; 1 = a little of the time; 2 = some of the time; 3 = a good bit of the time; 4 = most of the time; 5 = all of the time. The abbreviated HASTI score was the sum of these five parameters (range 0–25).

*Systemic Complication Variable Definitions*

Systemic complications included mild allergic reaction, severe allergic reaction, migraine, visual disturbance, cough/chest tightness, systemic infection, pulmonary embolism, transient ischemic attack, stroke, and/or death peri-procedurally. The definitions for these variables are derived from the Vascular Quality Initiative and are as follows:

- Mild allergic reaction: requires local treatment (i.e. topical steroids) or no treatment at all for symptoms like hives, rash, and/or itching
- Severe allergic reaction: requires systemic treatment for symptoms like difficulty breathing, difficulty swallowing, and/or anaphylaxis
- Migraine: intractable headache
- Visual disturbance: blurred vision, double vision, or other unusual change in vision, which is not related to a transient ischemic attack
- Cough/chest tightness: new symptoms of cough or chest tightness
- Systemic infection: infection in the bloodstream affecting multiple body systems
- Pulmonary embolism: blood clot which travels from another part of the body to the lungs
- Transient ischemic attack: brief acute focal neurological dysfunction, lasting less than 24 hours, usually involving loss of visual, motor or sensory function
- Stroke: acute cerebrovascular accident, lasting longer than 24 hours, usually involving loss of visual, motor or sensory function
- Death: mortality documented as an outcome related to the procedure

*Hematological Complication Variable Definitions*

Hematological complications included deep vein thrombosis, superficial phlebitis, major bleeding requiring procedural intervention, and/or hematoma within three months of the procedure. The definitions for these variables are derived from the Vascular Quality Initiative and are as follows:

- Deep vein thrombosis: deep vein thrombosis of the treated leg
- Superficial phlebitis: phlebitis that is in a superficial vein under the surface of the skin
- Major bleeding requiring intervention: transfusion, interventional, or surgical intervention required to treat bleeding or bleeding complication
- Hematoma: accumulation of blood at or around the site of treatment causing symptoms

*Proximal Thrombus Extension and Endothermal Heat-Induced Thrombosis Variable Definitions*

Proximal thrombus extension is defined by the Vascular Quality Initiative as any extension of the treatment-induced thrombus proximal to originally treated location.

Endothermal heat-induced thrombosis (EHIT) is defined by the Vascular Quality Initiative as thrombus extending proximal to the initial thermal ablation treatment level based on duplex imaging, with the following class assignments:

- EHIT class I: thrombus that does not extend into deep vein
- EHIT class II: thrombus extending into deep vein, non-occlusive, < 50% cross sectional area
- EHIT class III: thrombus extending into deep vein, non-occlusive, >50% cross sectional area
- EHIT class IV: thrombus occlusive in the deep vein

*Dermatologic Complication Variable Definitions*

Dermatologic complications included skin blistering, paresthesia, pigmentation, treatment-induced ulcer, and/or wound infection within three months of the procedure. The definitions for these variables are derived from the Vascular Quality Initiative and are as follows:

- Skin blistering: blisters on the surface of the skin at or around the site of treatment
- Paresthesia: sensation of tingling, tickling, prickling, pricking, or burning of the skin causing discomfort to the patient
- Pigmentation: discoloration of the skin associated with treatment of the underlying vein
- Treatment-induced ulcer: new skin ulcer as a result of the treatment
- Wound infection: superficial infection that involves the skin or subcutaneous tissue of the incision, with at least one of the following:
  - purulent drainage from the superficial incision
  - organisms isolated from an aseptically obtained culture of fluid or tissue from the superficial incision
  - superficial incision deliberately opened by the surgeon with at least one sign or symptom of infection (i.e., pain or tenderness, localized swelling, redness, or heat) and the obtained culture was positive or was not done
  - diagnosis of superficial incisional infection by the surgeon or attending physician
